# The Association Between Depressive Symptoms and Insomnia in College Students in Qinghai Province: The Mediating Effect of Rumination

**DOI:** 10.3389/fpsyt.2021.751411

**Published:** 2021-10-20

**Authors:** Shuheng Xiao, Shuai Liu, Puxiao Zhang, Jia Yu, Huaihong A, Hui Wu, Fabin Zhang, Yulan Xiao, Naiben Ma, Xiuqin Zhang, Xiaoxia Ma, Junfeng Li, Xiaodun Wang, Xin Shao, Wenjing Liu, Xiaolin Zhang, Wei Wu, Lihua Wang, Rihan Wu, Yinglian He, Zeyu Xu, Luhao Chi, Shixu Du, Bin Zhang

**Affiliations:** ^1^Department of Psychiatry, Nanfang Hospital, Southern Medical University, Guangzhou, China; ^2^The Third People's Hospital of Qinghai Province, Xining, China; ^3^First Clinical Medical College, Xinjiang Medical University, Urumqi, China; ^4^The Third People's Hospital of Panzhihua, Panzhihua, China; ^5^Department of Student Affairs, Qinghai University, Xining, China; ^6^Office of the President, Qinghai Nationalities University, Xining, China; ^7^Department of Student Affairs, Qinghai Nationalities University, Xining, China; ^8^Mental Health Education Center, Qinghai Nationalities University, Xining, China; ^9^School of Economics and Trade, Hebei GEO University, Shijiazhuang, China; ^10^School of Economics and Management, Qinghai Nationalities University, Xining, China; ^11^School of Civil Engineering, Tianjin University, Tianjin, China; ^12^School of Civil and Traffic Engineering, Qinghai Nationalities University, Xining, China; ^13^School of Physics Science and Information Technology, Liaocheng University, Liaocheng, China; ^14^School of Physics and Electronic Information Engineering, Qinghai Normal University, Xining, China; ^15^Institute of Health Management, Southern Medical University, Guangzhou, China

**Keywords:** depressive symptoms, insomnia, college students, mediation effect, ruminative thinking

## Abstract

**Background:** This study investigates the mediating effect of rumination on the associations between depressive symptoms and insomnia.

**Methods:** This is a cross-sectional study. Insomnia Severity Index (ISI), Ruminant Response Scale (RRS) and Beck Depression Inventory (BDI) were determined in 12,178 college students in Qinghai province by a questionnaire network platform.

**Results:** The prevalence of insomnia was 38.6% in the participants. Insomnia symptoms [interquartile range: 6 (3, 9)], depressive symptoms [interquartile range: 5 (1, 9)], and rumination [interquartile range: 22 (20, 26)] were positively correlated (*r* = 0.25–0.46, *p* < 0.01). Mediation effect analysis showed that the depressive symptoms affected insomnia directly and indirectly. The direct effect and the indirect effect through rumination account for 92.4 and 7.6% of the total effect, respectively.

**Conclusion:** The study shows that insomnia, depressive symptoms, and rumination are related constructs in college students in Qinghai province. It demonstrates the direct effects and the rumination-mediated indirect effects between depressive symptoms and insomnia; the direct effects seem to be dominant.

## Introduction

For Sleep plays a critical role in health and well-being of a human. It is reported that various populations are experiencing sleep disturbance frequently and suffering from its consequences. In Chinese college students, the prevalence of sleep disturbances and insomnia are reported to be 25.7% (1) and 18.5% (2), respectively.

Depression in youth has been shown to be associated with distinct sleep dimensions, such as timing, duration, and quality (3). Shochat et al. reported that those students with fewest depressive symptoms had moderate sleep time, shorter sleep onset latencies, and fewer arousals (4). In addition, depression can exacerbate the severity of insomnia in college students (5). Depression has been shown to be the most important risk factor for insomnia (6, 7). However, it is unclear if a depressive mood can lead to insomnia directly or indirectly through some mediating factors.

There may be some mediating factors between depressive symptoms and insomnia (8), such as lying awake at night (9), cognitive inflexibility (10), worry (11), social support (12), and internet addiction (13). Rumination is considered a possible mediator in relation between the depression and insomnia. It was well-known that individuals who have chronic insomnia worry about a range of topics while in bed, including “What about tomorrow's work?” (14). Rumination tends to engage in preservative and non-constructive thoughts and negative reflection on the problems and feelings in the past or present. Although previous studies have focused almost exclusively on the role of anxiety in the development of insomnia, it appears that rumination is more critical for eliciting sleep difficulties (15). Rumination about adverse events and self-reflection have been shown to heighten the levels of physiologic arousal (16), lengthen the sleep latency (17), and decrease the sleep efficiency (17). A clinical study showed that a high level of depressive symptoms significantly predicted a higher level of rumination (18). However, only a few studies have explored the mediating effect of rumination on the association between depressive symptoms and insomnia (19, 20). It has been shown that ruminative thoughts mediated the relationship between depressive symptoms and insomnia in the population of healthy young adults (20) and adolescents (19).

It has been reported that the sleep quality of students from high altitude areas is worse than that of students from plain areas, and the sleep quality of the ethnic minority students is worse than that of the Han students (21, 22). Qinghai province is located in the Qinghai-Tibet Plateau, with an average altitude of more than 3,000 meters. Many ethnic minority groups live in Qinghai province. The population of the Tibetan, Hui, Tu, Mongolian and Salar ethic groups accounts for 47.7% of the total population in Qinghai province (23). There is a lack of research on the relationship between insomnia and depressive symptoms in the population in this multi-ethnic area in Qinghai-Tibet Plateau. In this study we investigated the association between insomnia and depressive symptoms, and determined the mediating effect of rumination on the association between depressive symptoms and insomnia in college students in Qinghai province.

## Materials and Methods

### Participants and Procedures

The study was approved by the Ethics Committee of Nanfang Hospital of Southern Medical University and the Third People's Hospital of Qinghai Province. The study was conducted in accordance with the Declaration of Helsinki. Students from three universities (Qinghai University, Qinghai Normal University, and Qinghai Nationalities University) in Qinghai province were recruited using convenient sampling in November 2019. The inclusion criteria were: (1) Undergraduate students studying in the above three universities; (2) Wechat users; and (3) students participated in the study voluntarily. The exclusion criteria were: students on the campuses of the three aforementioned universities located outside Qinghai Province.

We conducted a cross-sectional survey on the online platform of Questionnaire Star. The purpose and significance of the survey were introduced to all participants, and the participant consent form was obtained from all participants prior to the study. The data were recorded and stored automatically. To protect the respondents' privacy, the survey was conducted anonymously. We put a total of 33 parameters into the model, including 30 observed variables or indicator variables and 3 latent factors. The sample size was required to be 5 or 10 times of the parameter, or 15 times of the observed or indicative variable (24). The minimum sample size in our study was 450.

### Measures

#### Socio-Demographics and Lifestyle Practice

Data of socio-demographics were collected from all participants, including gender, age, grade, ethnicity (Han, Tibetan, Hui, Tu, Mongolian, Salar, etc.), body weight, height, household monthly income (range: <5,000 RMB/month, 5,000–10,000 RMB/month, 10,000–20,000 RMB/month or >20,000 RMB/month), and being an only child in the family or not. Lifestyle and health conditions included chronic medical conditions, alcohol drinking, study stress (no, mild, or high), and boarding or commuting during school days.

#### Insomnia

Insomnia Severity Index (ISI) was used to evaluate the severity of insomnia during the past 2 weeks for each participant, as described previously (25). ISI consists of seven items. Each item is rated on a 5-point Likert scale (0 = none, 1 = mild, 2 = moderate, 3 = severe, 4 = extremely severe), with higher total scores indicating more severe insomnia. The scoring system is defined as follows: <8: no insomnia symptoms; 8–14: mild insomnia; 15–21: moderate insomnia; and 22–28: severe insomnia. The Cronbach's alpha of ISI is 0.83, and the 2-week test-retest reliability is 0.79 in Chinese adolescents (25).

#### Rumination

The 10-item Ruminative Responses Scale (RRS-10) was used to assess the severity of rumination. The Ruminative Responses Scale (RRS) was compiled by Nolen-Hoeksema, to assess the severity of depressive rumination (26). Treynor et al. removed 12 depression-related items from the RRS and developed a simplified scale called RRS-10 (27). The RRS-10 contains two subscales that reflect pondering and brooding, using a 4-point Likert scale. The Cronbach's α coefficient of this scale in Chinese college students is 0.90, and the test-retest reliability is 0.82 (28).

#### Depressive Symptoms

Beck Depression Inventory (BDI) (29) was used to assess depressive symptoms over the past week. BDI includes 13 items, and each item is rated on a scale from 0 to 3 (0: none, 1: mild, 2: moderate, and 3: severe). The scoring system for depressive symptoms is as follows: 0–4: no depressive symptoms; 5–7: mild depressive symptoms; 8–15: moderate depressive symptoms, and ≥16: severe depressive symptoms. The BDI has been shown to be a valid tool for a college student population (30). It has been found that the split-half coefficient of BDI in Chinese samples is 0.879, and the Cronbach's alpha is 0.890. The whole scale and each item group of the scale have good validity (31).

### Statistical Analysis

Analysis of structural equation structure (AMOS, version 24.0) and SPSS 21.0 software were used to analyze the data. Continuous variables (scale scores) with non-normal distribution are reported as the median [Interquartile range [IQR]]. The variables with normal distribution are reported as mean and standard deviation (mean ± SD). Categorical variables are reported as percentages. The direction and degree of correlation among factors were analyzed using Spearman's correlation analyses. The Bootstrap method of bias correction was used to test the mediating effect, and the parameter was set at 5,000 times. For the indirect effect, significance was considered at the level of *p* < 0.05, when the confidence interval did not contain zero. In the structural equation modeling (SEM), full information maximum likelihood was used to confirm interrelationships and parameters among the variables. The overall model fit was evaluated by using the likelihood ratio (χ2/df), the goodness of fit index (GFI), the Tucker-Lewis index and root (TLI), the comparative fit index (CFI), the root mean square error of approximation (RMSEA), and the root mean square residual (RMR). The Chi square (χ^2^) statistic was non-significant in the model, but it was highly influenced by sample size (32). Values of RMSEA ≤ 0.08 and RMR ≤ 0.05 were considered adequate model fit. The rest of the indices (e.g., GFI, TLI and CFI) ≥ 0.90 are indicative of an adequate model fit (33, 34).

## Results

### Demographic Characteristics of Participants

The demographic characteristics of participants are shown in Table 1. A total of 13,075 questionnaires were collected, and 12,178 (93.1%) participants responded with valid data. Among them, 8,065 participants (66.2%) were female. The mean age of participants was 20.2 ± 1.5 (range 16–30) years. The Han and Tibetan ethnic groups are the majority of the participants, accounting for 51.5% and 23.5% of the total population, respectively.

**Table 1:** Socio-demographic characteristics, health condition, mental distress and lifestyle practice of college students in Qinghai province (*N* = 12,178).

**Socio-demographics**	***N* (%) or Mean ± SD**
Age (years)	20.2 ± 1.5
BMI (kg/m^2^)	20.3 ± 2.5
**Gender**
Male	4,113 (33.8)
Female	8,065 (66.2)
**Ethnicity**
Han	6,258 (51.5)
Tibetan	2,866 (23.5)
Hui	1,730 (14.2)
Tu	674 (5.5)
Mongolian	322 (2.6)
Salar	108 (0.9)
Others	220 (1.8)
**Grade**
Freshman	5,705 (46.8)
Sophomore	3,549 (29.1)
Junior	1,984 (16.3)
Senior	940 (7.7)
Only child (Yes)	2,697 (22.1)
**Family income**
<¥5,000/month	7,639 (62.7)
¥5,000–10,000/month	3,404 (28)
¥10,000–20,000/month	903 (7.4)
>¥20,000/month	292 (1.9)
**Lifestyle practice and health conditions**
Alcohol use (Yes)	3,748 (30.8)
Chronic medical conditions (Yes)	321 (2.6)
High study stress (Yes)	2,935 (24.1)
Boarding in school (Yes)	12,099 (99.4)

*One Chinese Yuan (¥) equals 0.15 US Dollar. BMI, Body mass index*.

### Descriptive Statistics and Bivariate Correlations Between Measured Variables

The cross-sectional associations of insomnia, depressive symptoms, and rumination are presented in Table 2. The insomnia symptoms measured by the ISI scale revealed a sample median (IQR) score of 6 (3, 9). A total of 4,703 (38.6%) participants were considered insomniacs. Based on the BDI total score, a total of 6,250 participants (51.3%) suffered from depressive symptoms over the past week. Insomnia symptoms were significantly and positively correlated to depressive symptoms, brooding, and reflective pondering (*p* < 0.01), respectively.

**Table 2:** Descriptive statistics and bivariate correlations between insomnia symptoms, depressive symptoms, and rumination (*N* = 12,178).

	**Median (quartile)**	**ISI Total score**	**BDI Total score**	**RRS Total score**	**RRS Brooding**	**RRS Reflective pondering**
ISI total score	6 (3, 9)	1	–	–	–	–
BDI total score	5 (1, 9)	0.46^*^	1	–	–	–
RRS total score	22 (20, 26)	0.25^*^	0.36^*^	1	–	–
RRS-Brooding	11 (10, 14)	0.23^*^	0.37^*^	0.92^*^	1	–
RRS-Reflective pondering	10 (9, 13)	0.23^*^	0.30^*^	0.92^*^	0.72^*^	1

*ISI, Insomnia Severity Index; BDI, Beck Depression Inventory; RRS, Ruminative Responses Scale*.

* *Indicates significant correlation (p < 0.01)*.

### Structural Equation Modeling Results

**Figure 1:**
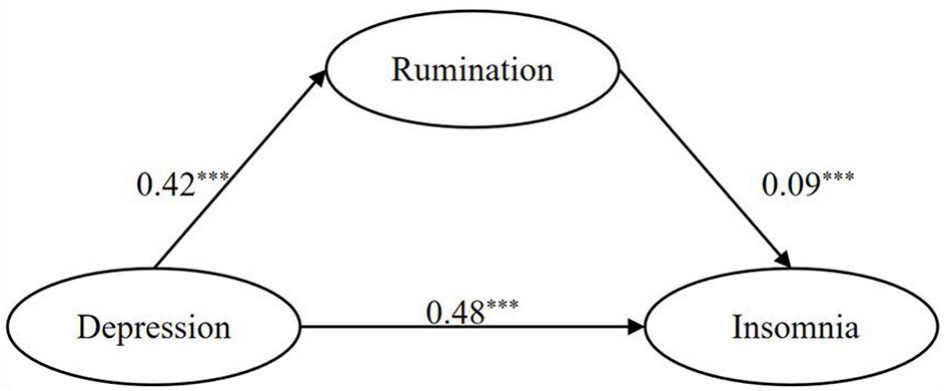
Model of mediating effect of rumination on insomnia symptoms and depressive symptoms. *** indicates path coefficients are significant (*p* < 0.05).

The mediating effect of rumination on the relationship between insomnia and depressive symptoms is described in Figure 1. Measurement models of latent variables were created for each measure of rumination and insomnia. The model fit was satisfactory for depressive symptoms, rumination, and insomnia (χ^2^/df = 35.215, RMSEA = 0.053, TLI = 0.903, GFI = 0.918, CFI = 0.910, RMR = 0.022).

The Bootstrap method with bias correction was used to test the mediating effect between insomnia symptoms and depressive symptoms. As shown in Table 3, in the groups of Han, Tibetan, Tu, Mongolian, the 95% confidence interval did not contain 0, indicating that an indirect effect existed in these groups. The indirect effect mediated by rumination appeared to be higher in the groups of Tibetan (10.8%), Tu (9.6%), and Mongolian (15.7%) compared to that of the Han group (5.8%). In the group of Hui and Salar, the 95% confidence interval of standardized indirect effect contained 0, indicating that an indirect effect did not existed. In Group Others (Table 3), including ethnic minorities Zhuang, Miao, and Man groups, the 95% confidence interval did not contain 0, indicating that an indirect effect existed; the indirect effect accounted for 16.7%. For the total samples, the 95% confidence interval of standardized indirect effect did not contain 0, indicating that an indirect effect existed. In the process from depressive symptoms to insomnia, the direct effect and indirect effect accounted for 92.4 and 7.6% of the total effect, respectively (Table 3).

**Table 3:** Bootstrap test of mediating effect of rumination between insomnia symptoms and depressive symptoms.

**Ethnic Groups**	**N**	**Pathways**	**Estimates**	**Standard Error**	**Bias-corrected CI (95%)**	
					**Lower**	**Upper**
						
Han	6258	Indirect effects	0.030 (5.8%)	0.006	0.019	0.042
		Direct effects	0.484 (94.2%)	0.015	0.454	0.514
		Total effects	0.514	0.013	0.488	0.539
						
Tibetan	2866	Indirect effects	0.054 (10.8%)	0.011	0.033	0.076
		Direct effects	0.448 (89.2%)	0.022	0.404	0.492
		Total effects	0.502	0.019	0.465	0.538
						
Hui	1730	Indirect effects	0.024 (4.7%)	0.016	−0.008	0.055
		Direct effects	0.491 (95.3%)	0.032	0.428	0.550
		Total effects	0.515	0.025	0.462	0.561
						
Tu	674	Indirect effects	0.048 (9.6%)	0.019	0.011	0.087
		Direct effects	0.451 (90.4%)	0.047	0.359	0.543
		Total effects	0.499	0.040	0.421	0.576
						
Mongolian	322	Indirect effects	0.081 (15.7%)	0.033	0.022	0.155
		Direct effects	0.434 (84.3%)	0.078	0.281	0.582
		Total effects	0.515	0.062	0.395	0.632
						
Salar	108	Indirect effects	0.087 (17.4%)	0.095	−0.020	0.324
		Direct effects	0.414 (82.6%)	0.140	0.099	0.653
		Total effects	0.501	0.106	0.292	0.704
						
Others	220	Indirect effects	0.098 (16.7%)	0.036	0.037	0.179
		Direct effects	0.489 (83.3%)	0.085	0.325	0.656
		Total effects	0.587	0.077	0.426	0.726
						
Total	12178	Indirect effects	0.039 (7.6%)	0.005	0.030	0.049
		Direct effects	0.476 (92.4%)	0.011	0.455	0.498
		Total effects	0.515	0.009	0.497	0.533

## Discussion

Our study showed that rumination mediated in the association between depressive symptoms and insomnia in college students in Qinghai province, a multi-ethnic and high-altitude area. However, the mediating impact is small.

In our study, 38.6% of college students reported that they suffered from insomnia in the past 2 weeks. Luo et al. demonstrated that the prevalence of insomnia among Chinese teenager in Guangzhou (average altitude: 21 meters) was 28.9% (35), which was lower than what we found. One reason may explain the difference is that nearly half of the participants in our study were ethnic minorities and lived in area with an average altitude of more than 3,000 meters. It has been shown that people who live at high altitudes have poorer sleep quality, which may be due to hypoxia-induced arousal and hypoxia-induced periodic breathing (35–37). Yip and Cheon reported that the prevalence of insomnia was high in ethnic minorities, which may be related to acculturation (38).

The results of the structural equation model display that depressive symptoms directly affect insomnia, and the direct effect accounted for 92.4% of the total effect. The results are consistent with previous studies that linking depressive symptoms to insomnia (8, 39). The youth with depressive symptoms demonstrate increased activity in extended medial network regions (40). Studies have shown that people with depressive symptoms have increased cortisol levels (41), hypothalamic pituitary adrenal (HPA) axis dysregulation (41), changes in inflammatory cytokines (42), and changes in sleep architecture (43). These changes can lead to insomnia. Beck's cognitive model of depression describes how people's thoughts and perceptions influence their emotional, behavioral, and physiological reactions. The elements of this model include biased attention, biased processing, biased thoughts and rumination, biased memory, and dysfunctional attitudes and schemas (44). These psychological processes may extend to the pre-sleep period, resulting in unpleasant intrusive thoughts, dysfunctional beliefs and attitudes about sleep, selective attention and monitoring to sleep-related threat, and misperception of sleep deficit. They may cause difficulty in initiating sleep and delayed sleep phase (45). Depressive symptoms are characterized by social withdrawal (46), poor interpersonal skills (47) and difficulty in coping with peer/family stressors (48). Such vigilance following social stressors may induce wakefulness before asleep.

In our study the structural equation model results display that rumination had mediating effects on the association between depressive symptoms and insomnia. Three possible mechanisms may explain our results. Firstly, depressive symptoms are considered the failure of emotion regulation. People with depressive symptoms may elaborate negative information. The reactivation of these memories during the pre-sleep period (i.e., ruminating) may exacerbate the difficulty in falling asleep (49). Secondly, ruminative thinking is considered invasive thinking. Individuals with excess rumination tend to focus on negative emotions continuously and repeatedly. This may further increase their selective attention to adverse events (50) and stimulate more cognitive awakening, leading to the delay of sleep initiation (8). Thirdly, rumination can cause psychological arousal and autonomic excitation, such as elevated heart rate, elevated body temperature, increased basal metabolic rate and electrodermal activity (51). Such changes in the body may cause insomnia.

Our results are consistent with previous studies (19, 20, 52) showing that rumination is a mediating factor between depressive symptoms and insomnia. However, there are some differences between our results and others. Firstly, the mediating effect by rumination in our study is smaller than that in the previous studies. Previous studies have reported that rumination fully mediated the relationship between depression and insomnia (19, 20). However, we found that rumination only plays a relatively limited role on the association between depressive symptom and insomnia. Secondly, previous studies included some other mediators, such as perfectionism (19), neuroticism (52), and self-reported health (20). Incorporating other factors into the model may change the indirect effects. Thirdly, about half of the participants in our study were from multiple ethnic minority groups. It has been shown that ruminative thinking from different cultural backgrounds is bound to have differences in content, form and function (53). For example, the everyday worship and annual Ramadan of the Hui Muslim group, including meditation and social networking, may promote psycho-physical well-being (54).

The present study has several strengths. Firstly, we applied a complex structural equation model to perform path analysis and quantify these paths. Secondly, the large sample size is a highlight of our study. Thirdly, the present study is the first sleep study conducted in the population of Qinghai-Tibet Plateau. It is also one of the few large-scale surveys conducted in the minority/multi-ethnic areas. However, several limitations should be noted when interpreting our findings. Firstly, due to the cross-sectional nature of the study, we cannot determine the causality among depressive symptoms, rumination, and insomnia. A longitudinal study will be designed to define the causality of these variables. Secondly, although rumination mediated the association between depressive symptoms and insomnia, the contribution of its effect was much less than that of the direct effect. Other unmeasured variables, such as attentional biases, self-control or social support (12), may play a role on the relationships among depressive symptoms, rumination, and insomnia. Thirdly, all the questionnaires in this study were self-reported. Although the self-report questionnaires provide a rapid access to a large number of college students' mental health status data via the Internet (45), the data may be prone to social desirability bias (55).

In summary, this study shows that insomnia, depressive symptoms, and rumination are related constructs in college students in Qinghai province. It demonstrates the direct effects and the rumination-mediated indirect effects between depressive symptoms and insomnia; the direct effects seem to be dominant.

## Data Availability Statement

The raw data supporting the conclusions of this article will be made available by the authors, without undue reservation.

## Ethics Statement

The studies involving human participants were reviewed and approved by Ethics Committee of Nanfang Hospital of Southern Medical University and the Third People's Hospital of Qinghai Province. Written informed consent to participate in this study was provided by the participants' legal guardian/next of kin.

## Author Contributions

SX and BZ: conceptualization. SX, ZX, and BZ: methodology. SX: writing—original draft. SX and SL: formal analysis. SL, PZ, JY, HA, HW, FZ, YX, NM, XiuZ, XM, JL, XW, XS, WL, XiaZ, WW, LW, RW, YH, LC, and BZ: investigation and resources. SL and BZ: writing—review and editing. PZ, JY, HA, HW, FZ, YX, NM, XiuZ, XM, JL, XW, XS, WL, XiaZ, WW, LW, RW, YH, LC, and SD: data curation. SL and BZ: funding acquisition. BZ: supervision and project administration. All authors have approved the final manuscript.

## Funding

This work was supported by the President Foundation of Nanfang Hospital, Southern Medical University (Grant No. 2019Z014); the Scientific Research Foundation of Southern Medical University (Grant No. CX2018N018); the National Natural Science Foundation of China (Grant No. 81901348); the Chinese Sleep Research Society Hansoh Project, China (Grant No. 2019HSC03); and the National Natural Science Foundation of China (Grant No. 82071488).

## Conflict of Interest

The authors declare that the research was conducted in the absence of any commercial or financial relationships that could be construed as a potential conflict of interest.

## Publisher's Note

All claims expressed in this article are solely those of the authors and do not necessarily represent those of their affiliated organizations, or those of the publisher, the editors and the reviewers. Any product that may be evaluated in this article, or claim that may be made by its manufacturer, is not guaranteed or endorsed by the publisher.
